# Characterizing Fibroblast Heterogeneity in Diabetic Wounds Through Single-Cell RNA-Sequencing

**DOI:** 10.3390/biomedicines12112538

**Published:** 2024-11-07

**Authors:** Helen H. Wang, Maria Korah, Serena L. Jing, Charlotte E. Berry, Michelle F. Griffin, Michael T. Longaker, Michael Januszyk

**Affiliations:** 1 Hagey Laboratory for Pediatric Regenerative Medicine, Division of Plastic and Reconstructive Surgery, Stanford University School of Medicine, Stanford, CA 94305, USA; wanghh@stanford.edu (H.H.W.); mkorah@stanford.edu (M.K.); sljing98@stanford.edu (S.L.J.); berryc@stanford.edu (C.E.B.); mgriff12@stanford.edu (M.F.G.); 2 Department of Surgery, Stanford University School of Medicine, Stanford, CA 94305, USA

**Keywords:** scRNAseq, single cell, transcriptomics, fibroblast, fibrosis, diabetes, wound healing

## Abstract

Diabetes mellitus is an increasingly prevalent chronic metabolic disorder characterized by physiologic hyperglycemia that, when left uncontrolled, can lead to significant complications in multiple organs. Diabetic wounds are common in the general population, yet the underlying mechanism of impaired healing in such wounds remains unclear. Single-cell RNA-sequencing (scRNAseq) has recently emerged as a tool to study the gene expression of heterogeneous cell populations in skin wounds. Herein, we review the history of scRNAseq and its application to the study of diabetic wound healing, focusing on how innovations in single-cell sequencing have transformed strategies for fibroblast analysis. We summarize recent research on the role of fibroblasts in diabetic wound healing and describe the functional and cellular heterogeneity of skin fibroblasts. Moreover, we highlight future opportunities in diabetic wound fibroblast research, with a focus on characterizing distinct fibroblast subpopulations and their lineages. Leveraging single-cell technologies to explore fibroblast heterogeneity and the complex biology of diabetic wounds may reveal new therapeutic targets for improving wound healing and ultimately alleviate the clinical burden of chronic wounds.

## 1. Introduction

Diabetes mellitus (DM), a metabolic disorder characterized by chronic hyperglycemia, is rapidly increasing in prevalence on a global scale. One recent study estimated that 529 million people worldwide were living with diabetes in 2021 [1]. The global prevalence of diabetes is expected to grow to 1.31 billion by 2050 due to trends in obesity and aging populations [1], posing an ever-increasing burden as chronic uncontrolled diabetes can lead to serious damage to multiple body systems [2]. Diabetic wounds are common and severe complications in patients with diabetes [3]. Such wounds are characterized by chronicity, slow healing, excessive inflammation, decreased angiogenesis, and poor scar quality [3]. Elevated blood glucose, peripheral neuropathy, and micro- and macrovascular complications impair wound healing capacity, leading to considerable morbidity and mortality [4]. Understanding the cell populations and mechanisms that promote normal regeneration is imperative for the development of effective interventions for impaired healing.

At a cellular level, the chief actuators that drive the wound healing process are fibroblasts. Fibroblasts have been extensively studied for their role in producing collagen and extracellular matrix (ECM) following injury, which accumulates in scar formation [5]. Recent studies have demonstrated that fibroblasts as a cell type are heterogeneous, and different subpopulations play different roles in the wound response [5,6,7]. For instance, reticular and hypodermal fibroblast lineages dominate the response to small wounds, while large wounds involve papillary fibroblasts and a subset of reticular-derived myofibroblasts [8,9]. The embryonic origins of fibroblast subpopulations may also influence cell-intrinsic fibrotic potential—neural crest-derived *WNT1* lineage-positive fibroblasts mediate scarless healing, while paraxial mesoderm-derived *EN1* lineage-positive fibroblasts and lateral plate mesoderm-derived *PRRX1* lineage-positive fibroblasts promote scarring [8,10,11]. Understanding how to modulate specific subpopulations could generate novel therapeutic approaches for chronic wounds.

Recent technological advances in single-cell RNA-sequencing (scRNAseq) methods have made the approach high throughput, widely adopted, and relatively affordable [12]. Importantly, scRNAseq can be used to delineate between cell types at a very high resolution and study rare cell populations such as cell subtypes and stem cell progenitors. Sequencing has benefited from rapid technological and methodological development due in part to the desirably amplifiable nature of DNA and RNA relative to other -omic measures of the cell. At present, challenges associated with applying scRNAseq to skin include noise in the data due to the small amount of starting RNA, higher fraction of dead cells and tissue, and potential unintended gene expression changes in response to sample processing steps, which are difficult for skin relative to other solid organs [13]. Chronic wounds, as found in diabetic skin, are particularly difficult to sequence due to the abundance of fibrinous debris and ECM and poor cell viability [14]. Nevertheless, continued improvements in the application of scRNAseq to diabetic wound healing are necessary to uncover novel insights into cellular heterogeneity, pathophysiological cell functions, and the intricate microenvironment of chronic wounds.

In this review, we examine every study that has attempted to perform scRNAseq on diabetic wound healing in humans or mice, outlining the historical development of scRNAseq technology and how its evolution has expanded our view of fibroblast heterogeneity within diabetic wound healing.

## 2. A History of Single Cell Transcriptomics

### 2.1. Precursors and the First Description of scRNAseq

In the early 1990s, efforts to study gene expression at a single-cell level were already underway. Eberwine et al. and Lambolez et al. each used single-cell RT-PCR to quantify the expression of a limited number of individual genes from single cells in 1992 [15,16]. Later PCR-based studies continued to increase the number of cells and genes interrogated [17], leading to the development of untargeted single-cell cDNA amplification techniques using microarrays [18,19]. The first example of true single-cell transcriptomics was demonstrated by Tang et al. in 2009, where they adapted an existing single-cell whole-transcriptome amplification method to be compatible with next-generation sequencing [20]. This allowed them to capture vastly more transcripts than other contemporary single-cell methods at the time and quantify their abundance in a single mouse blastomere. Following Tang et al., scRNAseq methods would accelerate in development, generating a variety of protocols that differed in their amplification technology, transcript coverage, extent of automation, and more.

### 2.2. Massively Parallel qPCR

Though scRNAseq arose in 2009, precursor methods like massively parallel qPCR were the first single-cell approaches to be applied to diabetic wound healing and continued to be used up to 2019 [21]. In 2014, Rennert et al. used a microfluidic-based single-cell approach to evaluate via qPCR approximately 70 gene targets in parallel per cell. Looking at genes relating to stemness, vasculogenesis, and tissue regeneration, they identified a subpopulation of adipose-derived stem cells characterized by high expression of angiogenic genes that were diminished in both type 1 and type 2 mouse models of diabetes [22]. Two years later, the same group developed a method to isolate phenotypically distinct progenitor cell subpopulations from microfluidic single-cell transcriptomic data and applied their platform to identify a subpopulation of progenitor cells that enhances wound healing potential in diabetic wounds [23]. The group continued to use microfluidic-based single-cell analysis in 2019 to assess the gene expression profiles of bone marrow-derived mesenchymal progenitor cells in diabetic wounds under different treatment conditions [24]. Collectively, these studies were early demonstrations of how single-cell transcriptomics could be used to isolate and characterize subpopulations of cells involved in wound healing.

### 2.3. Plate-Based Sequencing

The first scRNAseq experiment conducted by Tang et al. was limited in that it lacked technical controls that could separate the technical variation caused by amplification protocols from the true biological variation between cells, it preferentially amplified the 3′ ends of mRNA, and it only sequenced a single cell [20]. In 2011, Islam et al. described the first example of plate-based scRNAseq, single-cell tagged reverse transcription (STRT), which sequenced the whole transcriptomes of 96 single cells in a 96-well plate. STRT was able to use a barcoding approach to achieve multiplexed sequencing, but it only sequenced the 5′ ends of mRNA and did not generate read coverage across full transcripts [25,26]. In 2012, Ramsköld et al. introduced Smart-Seq, a plate-based method with markedly improved transcriptome coverage and the capability to sequence full-length reads, which could aid in the detection of transcript variants [27]. Plate-based methods like STRT and Smart-Seq achieved whole-transcriptome sequencing for large numbers of cells in parallel, leading to increased throughput and efficiency, reduced cost, and more comprehensive, unbiased, and high-resolution analysis of gene expression in single-cell experiments.

Though several different plate-based scRNAseq platforms were developed soon after Smart-Seq, such techniques were not applied to study diabetic wound healing until a decade later. Several studies used the BD Rhapsody™ Single-Cell Analysis System, which uses a cartridge workflow and barcoding system [28], to study the transcriptional profiles of cell subpopulations in diabetic wounds [29,30,31]. In one such study, He et al. investigated the heterogeneity of CD34+ cells in different wound models including diabetic wounds and identified 5 distinct clusters of fibroblasts. The abundances of each of these fibroblast clusters differed between diabetic and normal wounds, suggesting that fibroblast subpopulations held specific roles in diabetic and normal wound healing [30].

### 2.4. Droplet-Based Methods

Perhaps the most disruptive advancement in scRNAseq technologies was the invention of microdroplet-based isolation of single cells in 2015, when Macosko et al. introduced Drop-seq [32]. Up to this point, a major challenge for single-cell sequencing was the inability to quickly and easily isolate more than tens or hundreds of cells for ease of use and scale. Drop-seq achieved this in an automated fashion by combining a flow of cellular suspensions within reagents with timed addition of oil at set intervals to create nanoliter droplet emulsions [32]. Drop-seq could profile tens of thousands of cells, dramatically increasing the speed and scale at which complex tissues could be characterized [32]. This established the technological foundation for the now ubiquitous 10x Genomics Chromium Single Cell Gene Expression platform. According to the manufacturer’s publications database, as of the time of writing of this article, the Chromium platform has been used in over 6000 peer-reviewed publications since its release in 2016 [33], fueling the exponential growth of single-cell science.

Droplet-based scRNAseq has been rapidly adopted in diabetic wound healing research. Since 2020, the 10x Chromium platform has been used in 12 published diabetic wound healing studies [4,34,35,36,37,38,39,40,41,42,43,44] and 1 preprint [45], and a separate platform, SeekOne Digital Droplet Single Cell 3′ Transcriptome-seq, has been used in 1 study [46]. These methods have primarily generated discoveries about the roles of immune cells [34,35,36,37,38,39,40,41,46], fibroblasts [4,39,42,44,46], and keratinocytes [43] in diabetic wounds. Here, we will focus our discussion on findings related to fibroblasts.

Januszyk et al. generated a single-cell expression dataset from diabetic and non-diabetic plantar foot ulcers in the first detailed application of scRNAseq in human diabetic wound tissue. In their analysis, they identified fibroblast subpopulations with distinct fibrotic potentials, whose distributions differed between diabetic and non-diabetic cells [4]. Similarly, Theocharidis et al. generated a single-cell expression dataset from foot dorsum skin from subjects without DM, with DM without diabetic foot ulcers (DFU), and with DFU. They identified multiple fibroblast subpopulations, including a *COL7A1*-expressing population enriched specifically in DM skin, and further functional analysis suggested that chronic exposure to inflammation and hyperglycemia in DM skin incurs an injury response-associated gene expression profile in DM fibroblasts [42]. Two years later in 2022, Theocharidis et al. broadened their examination and profiled cells of the skin from DFU, foot, forearm, and peripheral blood mononuclear cell (PBMC) samples. They identified a distinct subpopulation of fibroblasts associated with wound healing that expressed multiple immune and ECM remodeling-related genes, suggesting that specific pro-healing fibroblast subtypes could be key targets for therapeutic development [39]. Most recently, the same group integrated their 2022 dataset with a new scRNAseq DFU dataset to conduct a race-focused analysis of healing-associated genes in DFU [44]. Their analysis showed distinct transcriptional differences in fibroblasts across racial groups, with lower expression of healing-associated genes in non-Hispanic Black patients compared to White patients, which they hypothesized reflects underlying disparities in environmental exposures and access to care.

In 2024, Wei et al. used CellChat, a computational tool used to infer intercellular signaling patterns [47], to show in scRNAseq data from skin wounds of a mouse DM model that fibroblasts expressed numerous ligands and receptors that interacted with immune cells [46]. Fibroblasts were enriched in the skin wounds of diabetic mice treated with a Janus liposozyme aimed at restoring tissue redox and immune homeostasis, indicating a potential immune cell-driven wound healing response.

### 2.5. Genomic Data Sharing: Reexamining Publicly Available Datasets

Public data repositories like Gene Expression Omnibus (GEO) have made it possible for previously generated datasets to be shared, reanalyzed, and integrated with other existing datasets. In 2018, the NIH updated its policy for genomic data sharing, with the goal of fostering meta-analyses on genomics data [48]. For scRNAseq, dataset integration can dramatically increase the accuracy, precision, and replicability of data analyses, mitigating experimental limitations such as cell count [49]. Moreover, reanalysis of publicly available data can apply newly developed bioinformatic approaches, that may not have existed at the time of the dataset’s generation or that the dataset authors may have lacked the expertise to execute. Reanalysis can also focus on a subset of the data that was not explored in depth by the dataset authors in order to unearth novel biological findings. Improved data sharing and rapidly developing biocomputational methods have ushered in the era of the meta-analysis, where data from multiple studies can be statistically combined to more powerfully interrogate biological research questions. One significant caveat of combining unique studies is that differences in technique can lead to unexpected alterations in sequencing results [50], necessitating robust dataset curation and integration methods [48].

In 2020, Davis et al. published a scRNAseq dataset of 4 samples of skin wounds and 4 samples of normal skin from diabetic patients [34]. This data was then reanalyzed in 2022 by Ou et al., who chose to focus their analysis on neural crest-derived cells and their interactions with other cell types. They found that Schwann cells displayed dysfunctional dedifferentiation, a vital function of Schwann cells in wound healing, in diabetic skin wounds [51]. Most recently, Ku et al. reanalyzed this dataset with a focus on fibroblasts [52]. Their investigation revealed dysregulation of the IGF-1-SP1-CD248 pathway in diabetic wounds, which may impair fibroblast migration and delay wound healing.

The most utilized scRNAseq dataset of diabetic wounds is that provided by Theocharidis et al. in 2022 [39]. This dataset contains scRNAseq profiles of foot and forearm skin cells, as well as PBMCs, from 10 non-diabetic subjects and 17 diabetic patients, 11 with and 6 without DFU. Eight publications have analyzed this publicly available dataset [53,54,55,56,57,58,59,60], often in combination with bulk transcriptomic data [53,54,55,56]. Of these studies, only 3 included analysis of fibroblast populations in the dataset [53,57,59], while the remainder focused on immune [54,58,60], endothelial [55,57], or stem cells [56]. Wang et al. found that fibroblasts and endothelial cells signaled strongly to each other during diabetic wound healing, and that both cell types, along with T cells and tissue stem cells, were closely associated with CCL signaling, which was more highly expressed in healing DFU tissues than in non-healing DFU tissues [59]. Lu et al. similarly compared healing DFUs and non-healing DFUs, and showed that healing-enriched fibroblasts (*DCN*+, *CHI3L1*+) were far more abundant in healing DFU than in non-healing DFU or healthy non-diabetic skin [57]. Chen and Zou distinguished distinct fibroblast subpopulations between healing and non-healing DFUs that exhibited different functions [53]. Healing-associated fibroblasts demonstrated increased ECM remodeling and a robust wound healing response, while non-healing-associated fibroblasts showed signs of cellular senescence and complement activation. Taken together, these studies indicate that dermal fibroblasts are heterogeneous with unique functional roles in wound healing, and specific fibroblast subtypes may be key to therapeutic interventions for DFU healing.

Most recently, Sandoval-Schaefer et al. released a scRNAseq dataset containing profiles of chronic diabetic foot ulcers and chronic non-diabetic foot ulcers as part of a preprint [45]. Subsequently, Chen et al. reanalyzed this single-cell data to identify marker genes specific to macrophage subpopulations in DFU, which they intersected with differentially expressed genes between DFU and normal samples and further analyzed to detect potentially valuable biomarkers associated with macrophages in DFU [61].

### 2.6. Comparing scRNAseq Approaches

In total, 30 studies have thus far applied scRNAseq to the study of diabetic wounds (Table 1). Though there are multiple platforms available within each of the approach categories we have outlined here, with some variability between them, broad comparisons can be made across these categories to highlight their advantages and disadvantages (Table 2). We use the metrics of read length, ischemia time between tissue harvest and sequencing, transcript coverage, and throughput to compare these approaches.

Read length refers to how much of the RNA molecule is captured in the sequencing read. A “full length” read refers to a sequencing read that captures the entire length of a molecule, while a “3′ end” or “5′ end” read only captures a single end of the molecule before reverse transcribing a subset of the full-length transcript, typically 50 base pairs. Full length scRNAseq methods are desirable for isoform analysis, allelic expression detection, RNA editing identification, and detection of lowly expressed genes or transcripts [62]. Presently, only some plate-based methods provide full-length reads. Droplet-based methods use single-end reads to instead provide a larger throughput of cells and a lower sequencing cost per cell [62].

The length of time between tissue harvest and sequencing, referred to as ischemia time, can dramatically affect the viability and expression profiles of the cells being sequenced [5,63]. Any method that relies on cell sorting prior to sequencing will inevitably require a longer ischemia time. Therefore, the approach with the shortest ischemia time is unsorted droplet-based sequencing.

Early scRNAseq approaches were unable to sequence the full transcriptome, limited to only panels of gene targets in the tens or hundreds. Beginning with plate-based methods, whole-transcriptome coverage improved substantially [27] and became the standard of single-cell sequencing for subsequently developed approaches.

Finally, though the scale and throughput of scRNAseq improved progressively in the decade following its inception, scRNAseq did not truly become high-throughput until the invention of Drop-seq. At the time of its publication, Drop-seq delivered over a one-hundred-fold improvement in both time and cost relative to existing methods [32]. To date, droplet-based approaches remain the most high-throughput method to perform single-cell sequencing.

## 3. Techniques for Fibroblast Isolation

Up until the development of Drop-seq, single cells were isolated for sequencing using cell sorting. Magnetic-activated cell sorting (MACS) or fluorescence-activated cell sorting (FACS) could be used to isolate live cells and potentially enrich for a population of interest using lineage gating against known markers. In addition to selecting for purity and viability, cell sorting also provided real-time phenotypic characterization using surface markers, ensuring that the sorted cells truly represented the desired functional subtype. However, cell sorting with FACS is costly, labor-intensive, and traumatic to cells, and is associated with decreased cell yield, increased time between cell isolation and droplet capture, and potential introduction of contaminants; with MACS these drawbacks are less pronounced, but still present. Preparatory steps in cell culture can lead to shifts in cell surface marker expression and even changes in cellular identity [5]. Importantly, sorting based on known markers introduces bias against cell populations that are understudied, poorly defined, or heterogeneous.

Cell sorting has now become an optional preparation step to help remove dead cells and debris or enrich for specific cell types of interest prior to sequencing. Specific cell types can now be “isolated” in silico, most commonly in a two-step process where cells are first clustered using unsupervised algorithms before being assigned cell type identities according to cluster-level expression profiles [64]. Not only is this computational analysis quickly implementable and highly scalable, but it also reduces cell loss when pre-sequencing sorting is omitted. With this in silico approach, depending on the resolution of the clustering and the gene factors selected to distinguish cell clusters from each other, cell type assignment and the granularity of such assignments can vary from study to study, despite using the same raw data. Importantly, as more markers are discovered to isolate specific cell populations, this approach can be used to apply updated definitions of cell populations to new or already existing sequencing data. This has enabled reanalyses of publicly available datasets to uncover novel information about heterogeneity within cell types.

A body of recent studies has contributed to a growing understanding of fibroblasts as a morphologically and functionally heterogeneous cell population [6]. In the isolation of fibroblasts for study, a persistent challenge has been the absence of a unified marker for this cell type. Though some markers are considered characteristic of fibroblasts, such as vimentin and collagen, these markers are considered nonspecific [5]. *PDGFRA* is often used as a “lineage marker,” yet not all fibroblasts express *PDGFRA* [65]. Thus, a common approach to identifying fibroblasts is that of exclusion, where fibroblasts are defined as cells that lack markers known to be specific to other cell types [8]. This exclusionary approach was first demonstrated by FACS in 2015 [66] and is now more easily performed in silico. Contemporary understanding of fibroblast heterogeneity and the continued lack of a clear genetic definition of this cell type suggest that earlier FACS-isolation strategies were inherently missing some subgroups of fibroblasts. With modern in silico characterization of fibroblasts, researchers can avoid the problem of subpopulation exclusion and study these subpopulations with increasing specificity.

## 4. Evolving Understanding of Fibroblast Heterogeneity

The evolution of single-cell sequencing has opened the door to more robust and unbiased mapping of fibroblast heterogeneity at previously impossible scale and resolution. ScRNAseq has been used to study fibroblast heterogeneity in human skin [67,68] and in murine skin wounds [69], parsing out spatial, functional, and lineage segregations. In 2021, Buechler et al. interrogated fibroblast heterogeneity across tissues by constructing a fibroblast atlas of single-cell transcriptomic data from 17 tissues, 50 datasets, 11 disease states and 2 species. They concluded that the fibroblast lineage could be divided into universal (*DPT*+) steady-state, specialized steady-state, and activated perturbation-specific cells. The authors suggested that these universal fibroblasts can differentiate into specialized and activated fibroblasts across diverse tissues. As such, this universal fibroblast population, which could be further subdivided into two *DPT*+ subtypes, may act as a resource of progenitor cells that provide functional plasticity to the fibroblast lineage [70]. This work not only established foundational organizing principles of the fibroblast lineage in health and disease, but also provided a resource that could facilitate identification and consensus definitions of fibroblast subtypes across tissues. To date, a similar study has not yet been conducted specifically for diabetic wounds.

Though fibroblast heterogeneity in diabetic wounds has not yet been studied at the depth of fibroblasts broadly, several studies have begun to uncover key fibroblast subpopulations in the diabetic wound response. Broadly, multiple studies have delineated pro-healing and non-healing fibroblasts as two different populations with distinct expression profiles.

Healing fibroblasts are associated with increased expression of ECM remodeling genes and immune signaling. Chen and Zou found that ECM remodeling is elevated in healing DFU relative to nonhealing, particularly in fibroblasts [53]. Moreover, matrix metalloproteinases (MMPs) and tissue inhibitors of matrix metalloproteinases (TMPs), which regulate ECM metabolism and remodeling, were widely downregulated in nonhealing DFU. Theocharidis et al. identified a unique population of fibroblasts overexpressing ECM remodeling (*MMP1*, *MMP3*, *MMP11*) and immune/inflammation (*HIF1A*, *CHI3L1*, *TNFAIP6*)-associated genes as a pro-healing population in DFU [39]. Choi et al. reinforced these findings in their study of fibroblasts of sharp debrided healing DFUs [44]. Several studies have pointed to an immune cell-driven wound healing response [39,46,59], where a subpopulation of fibroblasts capable of signaling with immune cells also promote ECM remodeling and improved wound healing [39,46]. Choi et al.’s race-focused analysis also highlights a plausible relationship between the reduced expression of key healing-associated genes such as *CHIL3L1*, *MMP11*, and *SFRP4* in healing fibroblasts of non-Hispanic Black (NHB) patients compared to White patients and the elevated occurrence of and poorer clinical outcomes of DFUs for NHBs compared to Whites [44].

For non-healing fibroblasts, Januszyk et al. identified two fibroblast subclusters with distinct pro-fibrotic gene expression profiles: one population exhibited upregulated *PTK2*, *PDGFRA*, and *DPP4*, while the other demonstrated elevated *JUN*, *FOS*, and *ACTA2*. They suggested that a small subpopulation of cells highly expressing *EN1* may drive the transcriptional shift into the latter pro-fibrotic state, identifying a therapeutic target [4]. Theocharidis et al. found that a *COL7A1*-expressing population of fibroblasts was specifically enriched in DM skin [42]. Excess type VII collagen in the dermis has previously been linked to scleroderma as a possible contributor to the tight and thick appearance of the affected skin [71]. Chen and Zou found that non-healing fibroblasts showed signs of cellular senescence and complement activation and that *APOD*, *APOE*, *COL3A1*, *PRG4*, and *TUBA1B* could serve as biomarkers for non-healing DFUs [53]. Lastly, Ku et al. underscore that dysregulation of the IGF1-SP1-CD248 axis in the fibroblasts of diabetic patients might cause impaired fibroblast migration and thus delayed wound healing, highlighting CD248 and SP1 as potential therapeutic targets to address diabetic ulcers [52].

## 5. Future Directions

Within the last 10 years, scRNAseq technologies have improved exponentially, stimulating a parallel explosion of interest in using single-cell sequencing to study biological questions. The future of scRNAseq is likely to see continued efforts to make single-cell sequencing more affordable on a per cell basis, an ever-increasing number of cells sequenced in a single study, improved transcript and isoform coverage, and a corresponding amassment of publicly available scRNAseq data. In response, we anticipate advances in bioinformatic and computational approaches to leverage the growing volume of data. This is already beginning to happen in the growth of published meta-analyses, as discussed above, and the development of interactive web apps to share and display datasets [72]. We hope that improvements in data storage and manipulation efficiency, computational power, and statistical methods will make analysis of these datasets easier and more accessible to a wider population of scientists. Finally, single-cell multi-omics is a growing trend that we expect to continue to gain traction, integrating scRNAseq with proteomic, epigenomic, and genomic data to better understand cell states and activities [73].

Empowered by rapidly advancing single-cell technologies, more studies remain to be conducted to understand fibroblasts and their roles in diabetic wounds. To isolate fibroblasts, either experimentally or in silico, further work is needed in the search for markers that define the cell type as a whole and its subpopulations. This information would aid fibroblast research broadly, generate signatures for disease diagnosis and prognosis, and deliver therapeutic targets.

Though Buechler et al. have established a foundational understanding of the fibroblast lineage, how fibroblasts achieve both general and specialized functions is unclear. Questions remain as to what intercellular signaling mechanisms and transcriptional or epigenetic programs stimulate differentiation from the universal type to specialized or activated states and what other subtypes might still be missing in existing data.

**Figure 1:**
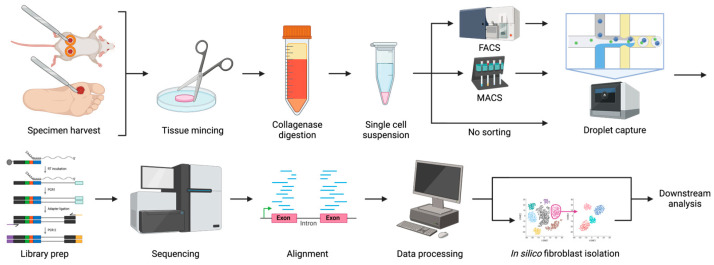
Illustrated Workflow for scRNAseq of Diabetic Wounds. An example workflow for single-cell RNA-seq of diabetic skin wounds using a droplet-based method such as 10x Genomics Chromium, from specimen harvest to downstream analysis of sequencing data. Optional cell sorting and in silico cell type isolation steps are illustrated using forked arrows. Created in BioRender. Wang, H. (2024) BioRender.com/p83k591 (accessed on 11 October 2024).

Generation of more modern scRNAseq datasets that are not biased by cell sorting is needed to supply a broad search space within which yet unidentified fibroblast subtypes might be uncovered and their lineage could be traced in detail. For diabetic wound healing, amassment of sufficient numbers of scRNAseq datasets would enable more powerful meta-analyses to identify fibroblast subtypes important for dysfunctional wound healing in diabetic skin. An example workflow for scRNAseq of diabetic wounds using a droplet-based method, highlighting optional steps for cell sorting and in silico fibroblast isolation, is shown in Figure 1.

The rise of spatial transcriptomics has also enabled the investigation of how fibroblast subgroups are dynamically spatially distributed. This has already been applied to dermal wound healing in mice [74]. However, spatial transcriptomic platforms such as Visium have not yet achieved single-cell resolution, as each “spot” from which transcripts are sequenced typically captures expression information from more than one cell [75]. This poses a challenge for the study of cell populations that may be closely related and only distinguishable by slight expression variation, such as subgroups of a cell type, since the average expression of a few cells in a spot might obscure the transcriptional signature that makes them unique. Deconvolution methods such as CytoSPACE have been developed to map individual cells from scRNAseq data to spatial expression profiles [76], yet because these methods inherently require both scRNAseq and spatial expression data, they impose additional cost and difficulty compared to an ideal single-cell-resolution spatial transcriptomic platform.

Ultimately, the goal of the research discussed here is to uncover the profiles and functions of cells, particularly fibroblasts, in diabetic wound healing, such that interventions may be developed. To date, no therapies specifically aimed at promoting wound repair, ameliorating scarring, or driving wound regeneration exist [8], let alone any designed specifically for diabetic wounds. Improved understanding of fibroblast heterogeneity and dynamics combined with translational studies in small and large animal models could deliver such therapies for clinical treatment of this debilitating condition.

## 6. Conclusions

The rapid evolution of single-cell transcriptomic sequencing since its advent 15 years ago has transformed our understanding of the diversity, plasticity, and function of fibroblasts in diabetic wound healing. Advancements in scRNAseq and increasingly abundant publicly available data have reinforced a growing view of fibroblasts as a highly heterogeneous cell type whose subpopulations can be modulated to promote healing or prevent disease. Fibroblasts can now be isolated and studied in silico in an unbiased fashion, rather than isolating them through traumatic cell sorting. For chronic wounds, which are at baseline more challenging than normal skin to sequence due to fibrinous content and poor cell viability, these methodological improvements have enabled an acceleration of study in this field in recent years. However, there is still much to be explored, including mechanisms of differentiation between fibroblast subtypes and other stromal and immune cells in and around the wound bed, detailed definition of the true extent of their heterogeneity, and mapping their spatial dynamics. Understanding the cell populations that orchestrate and act in diabetic wounds will deliver novel therapeutic interventions to reduce the burden of diabetic wounds.

## Figures and Tables

**Table 1:** scRNAseq Studies of Diabetic Wounds.

Title	Publication Year	PMID	scRNAseq Approach	scRNAseq Platform	Cell Types Sequenced
Diabetes Impairs the Angiogenic Potential of Adipose-Derived Stem Cells by Selectively Depleting Cellular Subpopulations	2014	24943716	Massively Parallel qPCR	Fluidigm Dynamic Array	Adipose-derived mesenchymal stem cells
Microfluidic Single-Cell Transcriptional Analysis Rationally Identifies Novel Surface Marker Profiles to Enhance Cell-Based Therapies	2016	27324848	Massively Parallel qPCR	Fluidigm Dynamic Array	Adipose-derived stem cells
Small Molecule Inhibition of Dipeptidyl Peptidase-4 Enhances Bone Marrow Progenitor Cell Function and Angiogenesis in Diabetic Wounds	2019	30452888	Massively Parallel qPCR	Fluidigm Dynamic Array	Bone marrow-derived mesenchymal progenitor cells
Characteristic Analysis of Skin Keratinocytes in Patients with Type 2 Diabetes Based on the Single-Cell Levels	2022	36583863	Plate-based Sequencing	BD Rhapsody	Endothelial cells, dendritic cells, fibroblasts, keratinocytes, mast cells, smooth muscle cells, T cells
Single-Cell Analysis Reveals Distinct Functional Heterogeneity of CD34+ Cells in Anagen Wound and Diabetic Wound	2023	36463761	Plate-based Sequencing	BD Rhapsody	Endothelial cells, fibroblasts, keratinocytes, macrophages, neutrophils, and T cells
Single-Cell RNA-Seq Analysis of Diabetic Wound Macrophages in STZ-Induced Mice	2023	36445632	Plate-based Sequencing	BD Rhapsody	CD45+ cells
Epigenetic Regulation of the PGE2 Pathway Modulates Macrophage Phenotype in Normal and Pathologic Wound Repair	2020	32879137	Droplet-based Methods	10x Genomics Chromium	B cells, basal cells, fibroblasts, interfollicular epidermal cells, macrophages, nerve cells, pericytes, T cells
Topical Application of a Mast Cell Stabilizer Improves Impaired Diabetic Wound Healing	2020	31568772	Droplet-based Methods	10x Genomics Chromium	Adipocytes, dendritic cells, endothelial cells, epithelial cells, fibroblasts, macrophages, mast cells, monocytes, neutrophils, smooth muscle cells, T cells
Enhanced Proliferation of Ly6C+ Monocytes/Macrophages Contributes to Chronic Inflammation in Skin Wounds of Diabetic Mice	2021	33443065	Droplet-based Methods	10x Genomics Chromium	Macrophages, monocytes
Reduced Apoptosis of Monocytes and Macrophages Is Associated with Their Persistence in Wounds of Diabetic Mice	2021	33810946	Droplet-based Methods	10x Genomics Chromium	Macrophages, monocytes
Macrophage-Specific Inhibition of the Histone Demethylase JMJD3 Decreases STING and Pathologic Inflammation in Diabetic Wound Repair	2022	36127466	Droplet-based Methods	10x Genomics Chromium	B cells, basal cells, interfollicular epidermal cells, fibroblasts, macrophages, nerve cells, pericytes, T cells
Single Cell Transcriptomic Landscape of Diabetic Foot Ulcers	2022	35013299	Droplet-based Methods	10x Genomics Chromium	B lymphocytes, dendritic/Langerhans cells, lymphatic endothelial cells, vascular endothelial cells, erythrocytes, fibroblasts, basal keratinocytes, differentiated keratinocytes, M1 macrophages, M2 macrophages, mast cells, melanocytes and Schwann cells, CD14+ monocytes, CD16+ monocytes, natural killer cells, NK and T cells, plasma cells, smooth muscle cells, sweat and sebaceous gland cells, T lymphocytes
Apoptosis Recognition Receptors Regulate Skin Tissue Repair in Mice	2023	38127424	Droplet-based Methods	10x Genomics Chromium	Dendritic cells, fibroblasts, macrophages, monocytes, neutrophils
Tracing Immunological Interaction in Trimethylamine N-Oxide Hydrogel-Derived Zwitterionic Microenvironment During Promoted Diabetic Wound Regeneration	2024	38885961	Droplet-based Methods	10x Genomics Chromium	B cell, endothelial cells, fibroblasts, basal keratinocytes, squamous keratinocytes, mast cells, macrophages, monocytes, neutrophils, T cells
Integrated Skin Transcriptomics and Serum Multiplex Assays Reveal Novel Mechanisms of Wound Healing in Diabetic Foot Ulcers	2020	32763913	Droplet-based Methods	10x Genomics Chromium	B cells, fibroblasts, lymphatic endothelial cells, vascular endothelial cells, macrophages, monocytes, smooth muscle cells, T cells
IFN-κ Is Critical for Normal Wound Repair and Is Decreased in Diabetic Wounds	2022	35358091	Droplet-based Methods	10x Genomics Chromium	B cells, basal cells, differentiated cells, fibroblasts, keratinized cells, macrophages, mast cells, nerve cells, pericytes, T cells
Transcriptional Heterogeneity in Human Diabetic Foot Wounds	2023	36824808	Droplet-based Methods	10x Genomics Chromium	B cells, endothelial cells, fibroblasts, keratinocytes, lymphatic cells, macrophages, mast cells, pericytes, T cells
Janus Liposozyme for the Modulation of Redox and Immune Homeostasis in Infected Diabetic Wounds	2024	38740936	Droplet-based Methods	SeekOne Digital Droplet	Adipocytes, dendritic cells, endothelial cells, fibroblasts, M1 macrophages, M2 macrophages, M2-like macrophages, mast cells, neutrophils, αβ T cells, γδ T cells
Single-cell Analysis of Debrided Diabetic Foot Ulcers Reveals Dysregulated Wound Healing Environment in non-Hispanic Blacks	2024	39127092	Droplet-based Methods	10x Genomics Chromium	B lymphocytes, dendritic/Langerhans cells, lymphatic endothelial cells, vascular endothelial cells, fibroblasts, angiogenic macrophages, inflammatory macrophages, mast cells, neutrophils, natural killer and T cells, plasma cells, smooth muscle cells, T lymphocytes
Dedifferentiated Schwann Cell-Derived TGF-Β3 Is Essential for the Neural System to Promote Wound Healing	2022	35910794	Meta-analyses	N/A	Epidermal and basal cells, fibroblasts, immune cells, neural crest-derived cells, vascular cells
Deciphering the Dysregulating IGF-1-SP1-CD248 Pathway in Fibroblast Functionality during Diabetic Wound Healing	2024	39293711	Meta-analyses	N/A	Fibroblasts, endothelial cells, keratinocytes, macrophages, mast cells, melanocytes, nerve cells, pericytes/smooth muscle cells, T cells
Combined Analysis of Single-Cell Sequencing and Bulk Transcriptome Sequencing Reveals New Mechanisms for Non-Healing Diabetic Foot Ulcers	2024	38950058	Meta-analyses	N/A	B lymphocytes, basal keratinocytes, lymphatic endothelial cells, vascular endothelial cells, fibroblasts, differentiated keratinocytes, lymphatic, M1 macrophages, M2 macrophages, mast cells, melanocytes and Schwann cells, Merkel cells, natural killer cells, plasma cells, smooth muscle cells, T lymphocytes
Identification of Potential Immunologic Resilience in the Healing Process of Diabetic Foot Ulcers	2024	37926487	Meta-analyses	N/A	Adipocytes, B cells, endothelial cells, epithelial cells, fibroblasts, hematopoietic stem cells, keratinocytes, macrophages, monocytes, melanocytes, CD8+ T cells
Single-Cell RNA-Seq and Bulk-Seq Identify RAB17 as a Potential Regulator of Angiogenesis by Human Dermal Microvascular Endothelial Cells in Diabetic Foot Ulcers	2023	37605780	Meta-analyses	N/A	B cells, basal keratinocytes, differentiated keratinocytes, dermal microvascular endothelial cells, fibroblasts, lymphatic endothelial cells, mast cells/melanocytes, monocytes/macrophages, neural cells, plasma cells, smooth muscle cells, T cells
Characterization of the Microenvironment of Diabetic Foot Ulcers and Potential Drug Identification Based on scRNA-Seq	2022	36686438	Meta-analyses	N/A	B cells, basal cells, endothelial cells, epithelial cells, granulosa cells, hematopoietic cells, leukocytes, macrophages, mast cells, monocytes, natural killer cells, plasma cells, pluripotent stem cells, stem cells, stromal cells, vascular smooth muscle cells
Single-Cell Profiling Reveals Transcriptomic Signatures of Vascular Endothelial Cells in Non-Healing Diabetic Foot Ulcers	2023	38107519	Meta-analyses	N/A	B lymphocytes, basal keratinocytes, lymphatic endothelial cells, vascular endothelial cells, fibroblasts, differentiated keratinocytes, M1 macrophages, M2 macrophages, mast cells, melanocytes and Schwann cells, NK cells and T lymphocytes, smooth muscle cells
San Huang Xiao Yan Recipe Modulates the HMGB1-Mediated Abnormal Inflammatory Microenvironment and Ameliorates Diabetic Foot by Activating the AMPK/Nrf2 Signalling Pathway	2023	37364421	Meta-analyses	N/A	M1 macrophages
Healing Mechanism of Diabetic Foot Ulcers Using Single-Cell RNA-Sequencing	2023	37007553	Meta-analyses	N/A	B cells, endothelial cells, epithelial cells, fibroblasts, keratinocytes, monocytes/macrophages, T cells, tissue stem cells
Comprehensive transcriptomic analysis of immune-related genes in diabetic foot ulcers: New insights into mechanisms and therapeutic targets	2024	39079197	Meta-analyses	N/A	B cells, endothelial cells, epithelial cells, fibroblasts, mast cells, monocytes/macrophages, plasma cells, smooth muscle cells, T cells
HMOX1 as a Therapeutic Target Associated with Diabetic Foot Ulcers Based on Single-Cell Analysis and Machine Learning	2024	38468410	Meta-analyses	N/A	Chondrocytes, common myeloid progenitor cells, endothelial cells, epithelial cells, fibroblasts, keratinocytes, macrophages, monocytes, T cells, tissue stem cells

Compilation of scRNAseq studies of diabetic wounds, with annotated scRNAseq approach and platform. The column “Cell Types Sequenced” lists the cell types isolated from sorting steps prior to sequencing, or if the study was unsorted, the cell types identified in silico from sequencing data.

**Table 2:** scRNAseq Approaches with Advantages and Disadvantages.

Technique	Full Length	Low Ischemia Time	Full Gene Coverage	High Throughput
Massively Parallel qPCR				
Plate-based Sequencing	✓ *		✓	
Droplet-based Methods (Unsorted)		✓	✓	✓
Droplet-based Methods (Sorted)			✓	✓

Summary comparison of scRNAseq approaches with notable advantages and disadvantages. A check mark indicates that the scRNAseq technique in the checked row is characterized by the feature in the checked column. * There are a wide variety of plate-based sequencing methods: some sequence full-length transcripts (e.g., Smart-seq) while others sequence 3′ (e.g., MARS-seq) or 5′-only (e.g., STRT-seq) [62].

## Data Availability

No new data were created.

## References

[1] Ong K.L., Stafford L.K., McLaughlin S.A., Boyko E.J., Vollset S.E., Smith A.E., Dalton B.E., Duprey J., Cruz J.A., Hagins H. (2023). Global, Regional, and National Burden of Diabetes from 1990 to 2021, with Projections of Prevalence to 2050: A Systematic Analysis for the Global Burden of Disease Study 2021. Lancet.

[2] Forbes J.M., Cooper M.E. (2013). Mechanisms of Diabetic Complications. Physiol. Rev..

[3] Dasari N., Jiang A., Skochdopole A., Chung J., Reece E.M., Vorstenbosch J., Winocour S. (2021). Updates in Diabetic Wound Healing, Inflammation, and Scarring. Semin. Plast. Surg..

[4] Januszyk M., Chen K., Henn D., Foster D.S., Borrelli M.R., Bonham C.A., Sivaraj D., Wagh D., Longaker M.T., Wan D.C. (2020). Characterization of Diabetic and Non-Diabetic Foot Ulcers Using Single-Cell RNA-Sequencing. Micromachines.

[5] Lynch M.D., Watt F.M. (2018). Fibroblast Heterogeneity: Implications for Human Disease. J. Clin. Investig..

[6] Parker J.B., Valencia C., Akras D., DiIorio S.E., Griffin M.F., Longaker M.T., Wan D.C. (2023). Understanding Fibroblast Heterogeneity in Form and Function. Biomedicines.

[7] Driskell R.R., Watt F.M. (2015). Understanding Fibroblast Heterogeneity in the Skin. Trends Cell Biol..

[8] Talbott H.E., Mascharak S., Griffin M., Wan D.C., Longaker M.T. (2022). Wound Healing, Fibroblast Heterogeneity, and Fibrosis. Cell Stem Cell.

[9] Driskell R.R., Lichtenberger B.M., Hoste E., Kretzschmar K., Simons B.D., Charalambous M., Ferron S.R., Herault Y., Pavlovic G., Ferguson-Smith A.C. (2013). Distinct Fibroblast Lineages Determine Dermal Architecture in Skin Development and Repair. Nature.

[10] Rinkevich Y., Walmsley G.G., Hu M.S., Maan Z.N., Newman A.M., Drukker M., Januszyk M., Krampitz G.W., Gurtner G.C., Lorenz H.P. (2015). Identification and Isolation of a Dermal Lineage with Intrinsic Fibrogenic Potential. Science.

[11] Mascharak S., desJardins-Park H.E., Davitt M.F., Griffin M., Borrelli M.R., Moore A.L., Chen K., Duoto B., Chinta M., Foster D.S. (2021). Preventing Engrailed-1 Activation in Fibroblasts Yields Wound Regeneration without Scarring. Science.

[12] Jovic D., Liang X., Zeng H., Lin L., Xu F., Luo Y. (2022). Single-cell RNA Sequencing Technologies and Applications: A Brief Overview. Clin. Transl. Med..

[13] Kim D., Chung K.B., Kim T.-G. (2020). Application of Single-Cell RNA Sequencing on Human Skin: Technical Evolution and Challenges. J. Dermatol. Sci..

[14] Januszyk M., Gurtner G.C. (2013). High-Throughput Single-Cell Analysis for Wound Healing Applications. Adv. Wound Care.

[15] Eberwine J., Yeh H., Miyashiro K., Cao Y., Nair S., Finnell R., Zettel M., Coleman P. (1992). Analysis of Gene Expression in Single Live Neurons. Proc. Natl. Acad. Sci. USA.

[16] Lambolez B., Audinat E., Bochet P., Crépel F., Rossier J. (1992). AMPA Receptor Subunits Expressed by Single Purkinje Cells. Neuron.

[17] Peixoto A., Monteiro M., Rocha B., Veiga-Fernandes H. (2004). Quantification of Multiple Gene Expression in Individual Cells. Genome Res..

[18] Kurimoto K., Yabuta Y., Ohinata Y., Ono Y., Uno K.D., Yamada R.G., Ueda H.R., Saitou M. (2006). An Improved Single-Cell cDNA Amplification Method for Efficient High-Density Oligonucleotide Microarray Analysis. Nucleic Acids Res..

[19] Kurimoto K., Yabuta Y., Ohinata Y., Saitou M. (2007). Global Single-Cell cDNA Amplification to Provide a Template for Representative High-Density Oligonucleotide Microarray Analysis. Nat. Protoc..

[20] Tang F., Barbacioru C., Wang Y., Nordman E., Lee C., Xu N., Wang X., Bodeau J., Tuch B.B., Siddiqui A. (2009). mRNA-Seq Whole-Transcriptome Analysis of a Single Cell. Nat. Methods.

[21] Januszyk M., Sorkin M., Glotzbach J.P., Vial I.N., Maan Z.N., Rennert R.C., Duscher D., Thangarajah H., Longaker M.T., Butte A.J. (2014). Diabetes Irreversibly Depletes Bone Marrow-Derived Mesenchymal Progenitor Cell Subpopulations. Diabetes.

[22] Rennert R.C., Sorkin M., Januszyk M., Duscher D., Kosaraju R., Chung M.T., Lennon J., Radiya-Dixit A., Raghvendra S., Maan Z.N. (2014). Diabetes Impairs the Angiogenic Potential of Adipose-Derived Stem Cells by Selectively Depleting Cellular Subpopulations. Stem Cell Res. Ther..

[23] Rennert R.C., Januszyk M., Sorkin M., Rodrigues M., Maan Z.N., Duscher D., Whittam A.J., Kosaraju R., Chung M.T., Paik K. (2016). Microfluidic Single-Cell Transcriptional Analysis Rationally Identifies Novel Surface Marker Profiles to Enhance Cell-Based Therapies. Nat. Commun..

[24] Whittam A.J., Maan Z.N., Duscher D., Barrera J.A., Hu M.S., Fischer L.H., Khong S., Kwon S.H., Wong V.W., Walmsley G.G. (2019). Small Molecule Inhibition of Dipeptidyl Peptidase-4 Enhances Bone Marrow Progenitor Cell Function and Angiogenesis in Diabetic Wounds. Transl. Res. J. Lab. Clin. Med..

[25] Islam S., Kjällquist U., Moliner A., Zajac P., Fan J.-B., Lönnerberg P., Linnarsson S. (2011). Characterization of the Single-Cell Transcriptional Landscape by Highly Multiplex RNA-Seq. Genome Res..

[26] Islam S., Kjällquist U., Moliner A., Zajac P., Fan J.-B., Lönnerberg P., Linnarsson S. (2012). Highly Multiplexed and Strand-Specific Single-Cell RNA 5′ End Sequencing. Nat. Protoc..

[27] Ramsköld D., Luo S., Wang Y.-C., Li R., Deng Q., Faridani O.R., Daniels G.A., Khrebtukova I., Loring J.F., Laurent L.C. (2012). Full-Length mRNA-Seq from Single-Cell Levels of RNA and Individual Circulating Tumor Cells. Nat. Biotechnol..

[28] Shum E.Y., Walczak E.M., Chang C., Christina Fan H. (2019). Quantitation of mRNA Transcripts and Proteins Using the BD Rhapsody^TM^ Single-Cell Analysis System. Adv. Exp. Med. Biol..

[29] Liao B., Ouyang Q., Song H., Wang Z., Ou J., Huang J., Liu L. (2022). Characteristic Analysis of Skin Keratinocytes in Patients with Type 2 Diabetes Based on the Single-Cell Levels. Chin. Med. J..

[30] He J., Huang W., Wang J., Li G., Xin Q., Lin Z., Chen X., Wang X. (2023). Single-Cell Analysis Reveals Distinct Functional Heterogeneity of CD34+ Cells in Anagen Wound and Diabetic Wound. Biochem. Biophys. Res. Commun..

[31] Ma J., Song R., Liu C., Cao G., Zhang G., Wu Z., Zhang H., Sun R., Chen A., Wang Y. (2023). Single-Cell RNA-Seq Analysis of Diabetic Wound Macrophages in STZ-Induced Mice. J. Cell Commun. Signal..

[32] Macosko E.Z., Basu A., Satija R., Nemesh J., Shekhar K., Goldman M., Tirosh I., Bialas A.R., Kamitaki N., Martersteck E.M. (2015). Highly Parallel Genome-Wide Expression Profiling of Individual Cells Using Nanoliter Droplets. Cell.

[33] Publications. https://www.10xgenomics.com/publications.

[34] Davis F.M., Tsoi L.C., Wasikowski R., denDekker A., Joshi A., Wilke C., Deng H., Wolf S., Obi A., Huang S. (2020). Epigenetic Regulation of the PGE2 Pathway Modulates Macrophage Phenotype in Normal and Pathologic Wound Repair. JCI Insight.

[35] Tellechea A., Bai S., Dangwal S., Theocharidis G., Nagai M., Koerner S., Cheong J.E., Bhasin S., Shih T.-Y., Zheng Y. (2020). Topical Application of a Mast Cell Stabilizer Improves Impaired Diabetic Wound Healing. J. Investig. Dermatol..

[36] Pang J., Maienschein-Cline M., Koh T.J. (2021). Enhanced Proliferation of Ly6C+ Monocytes/Macrophages Contributes to Chronic Inflammation in Skin Wounds of Diabetic Mice. J. Immunol..

[37] Pang J., Maienschein-Cline M., Koh T.J. (2021). Reduced Apoptosis of Monocytes and Macrophages Is Associated with Their Persistence in Wounds of Diabetic Mice. Cytokine.

[38] Audu C.O., Melvin W.J., Joshi A.D., Wolf S.J., Moon J.Y., Davis F.M., Barrett E.C., Mangum K.D., Deng H., Xing X. (2022). Macrophage-Specific Inhibition of the Histone Demethylase JMJD3 Decreases STING and Pathologic Inflammation in Diabetic Wound Repair. Cell. Mol. Immunol..

[39] Theocharidis G., Thomas B.E., Sarkar D., Mumme H.L., Pilcher W.J.R., Dwivedi B., Sandoval-Schaefer T., Sîrbulescu R.F., Kafanas A., Mezghani I. (2022). Single Cell Transcriptomic Landscape of Diabetic Foot Ulcers. Nat. Commun..

[40] Justynski O., Bridges K., Krause W., Forni M.F., Phan Q.M., Sandoval-Schaefer T., Carter K., King D.E., Hsia H.C., Gazes M.I. (2023). Apoptosis Recognition Receptors Regulate Skin Tissue Repair in Mice. eLife.

[41] Li Z., Li L., Yue M., Peng Q., Pu X., Zhou Y. (2024). Tracing Immunological Interaction in Trimethylamine N-Oxide Hydrogel-Derived Zwitterionic Microenvironment During Promoted Diabetic Wound Regeneration. Adv. Mater..

[42] Theocharidis G., Baltzis D., Roustit M., Tellechea A., Dangwal S., Khetani R.S., Shu B., Zhao W., Fu J., Bhasin S. (2020). Integrated Skin Transcriptomics and Serum Multiplex Assays Reveal Novel Mechanisms of Wound Healing in Diabetic Foot Ulcers. Diabetes.

[43] Wolf S.J., Audu C.O., Joshi A., denDekker A., Melvin W.J., Davis F.M., Xing X., Wasikowski R., Tsoi L.C., Kunkel S.L. (2022). IFN-κ Is Critical for Normal Wound Repair and Is Decreased in Diabetic Wounds. JCI Insight.

[44] Choi D., Bakhtiari M., Pilcher W., Huang C., Thomas B.E., Mumme H., Blanco G., Rajani R., Schechter M.C., Fayfman M. (2024). Single-Cell Analysis of Debrided Diabetic Foot Ulcers Reveals Dysregulated Wound Healing Environment in Non-Hispanic Blacks. J. Investig. Dermatol..

[45] Sandoval-Schaefer T., Phan Q., Dash B.C., Prassinos A.J., Duan K., Gazes M.I., Vyce S.D., Driskell R., Hsia H.C., Horsley V. (2023). Transcriptional Heterogeneity in Human Diabetic Foot Wounds. bioRxiv.

[46] Wei T., Pan T., Peng X., Zhang M., Guo R., Guo Y., Mei X., Zhang Y., Qi J., Dong F. (2024). Janus Liposozyme for the Modulation of Redox and Immune Homeostasis in Infected Diabetic Wounds. Nat. Nanotechnol..

[47] Jin S., Guerrero-Juarez C.F., Zhang L., Chang I., Ramos R., Kuan C.-H., Myung P., Plikus M.V., Nie Q. (2021). Inference and Analysis of Cell-Cell Communication Using CellChat. Nat. Commun..

[48] Psaty B.M., Rich S.S., Boerwinkle E. (2019). Innovation in Genomic Data Sharing at the NIH. N. Engl. J. Med..

[49] Rocque B., Barbetta A., Singh P., Goldbeck C., Helou D.G., Loh Y.-H.E., Ung N., Lee J., Akbari O., Emamaullee J. (2021). Creation of a Single Cell RNASeq Meta-Atlas to Define Human Liver Immune Homeostasis. Front. Immunol..

[50] Denisenko E., Guo B.B., Jones M., Hou R., de Kock L., Lassmann T., Poppe D., Clément O., Simmons R.K., Lister R. (2020). Systematic Assessment of Tissue Dissociation and Storage Biases in Single-Cell and Single-Nucleus RNA-Seq Workflows. Genome Biol..

[51] Ou M.-Y., Tan P.-C., Xie Y., Liu K., Gao Y.-M., Yang X.-S., Zhou S.-B., Li Q.-F. (2022). Dedifferentiated Schwann Cell-Derived TGF-Β3 Is Essential for the Neural System to Promote Wound Healing. Theranostics.

[52] Ku Y.-C., Lee Y.-C., Hong Y.-K., Lo Y.-L., Kuo C.-H., Wang K.-C., Hsu C.-K., Yu C.-H., Lin S.-W., Wu H.-L. (2024). Deciphering the Dysregulating IGF-1-SP1-CD248 Pathway in Fibroblast Functionality during Diabetic Wound Healing. J. Investig. Dermatol..

[53] Chen R., Zou L. (2024). Combined Analysis of Single-Cell Sequencing and Bulk Transcriptome Sequencing Reveals New Mechanisms for Non-Healing Diabetic Foot Ulcers. PLoS ONE.

[54] Cheng Y., Ren L., Niyazi A., Sheng L., Zhao Y. (2024). Identification of Potential Immunologic Resilience in the Healing Process of Diabetic Foot Ulcers. Int. Wound J..

[55] Du H., Li S., Lu J., Tang L., Jiang X., He X., Liang J., Liao X., Cui T., Huang Y. (2023). Single-Cell RNA-Seq and Bulk-Seq Identify RAB17 as a Potential Regulator of Angiogenesis by Human Dermal Microvascular Endothelial Cells in Diabetic Foot Ulcers. Burns Trauma.

[56] Li Y., Ju S., Li X., Li W., Zhou S., Wang G., Cai Y., Dong Z. (2022). Characterization of the Microenvironment of Diabetic Foot Ulcers and Potential Drug Identification Based on scRNA-Seq. Front. Endocrinol..

[57] Lu Y., Liu X., Zhao J., Bie F., Liu Y., Xie J., Wang P., Zhu J., Xiong Y., Qin S. (2023). Single-Cell Profiling Reveals Transcriptomic Signatures of Vascular Endothelial Cells in Non-Healing Diabetic Foot Ulcers. Front. Endocrinol..

[58] Zhang Z., Zheng Y., Chen N., Xu C., Deng J., Feng X., Liu W., Ma C., Chen J., Cai T. (2023). San Huang Xiao Yan Recipe Modulates the HMGB1-Mediated Abnormal Inflammatory Microenvironment and Ameliorates Diabetic Foot by Activating the AMPK/Nrf2 Signalling Pathway. Phytomed. Int. J. Phytother. Phytopharm..

[59] Wang Z., Wei D., Li S., Tang Q., Lu G., Gu S., Lu L., Liang F., Teng J., Lin J. (2023). Healing Mechanism of Diabetic Foot Ulcers Using Single-Cell RNA-Sequencing. Ann. Transl. Med..

[60] Jiang N., Xu C., Xu Y., Zhuo Y., Chen P., Deng S., Zhao Z., Long Y., Bai X., Wang Q. (2024). Comprehensive Transcriptomic Analysis of Immune-Related Genes in Diabetic Foot Ulcers: New Insights into Mechanisms and Therapeutic Targets. Int. Immunopharmacol..

[61] Chen Y., Zhang Y., Jiang M., Ma H., Cai Y. (2024). HMOX1 as a Therapeutic Target Associated with Diabetic Foot Ulcers Based on Single-Cell Analysis and Machine Learning. Int. Wound J..

[62] Chen G., Ning B., Shi T. (2019). Single-Cell RNA-Seq Technologies and Related Computational Data Analysis. Front. Genet..

[63] Massoni-Badosa R., Iacono G., Moutinho C., Kulis M., Palau N., Marchese D., Rodríguez-Ubreva J., Ballestar E., Rodriguez-Esteban G., Marsal S. (2020). Sampling Time-Dependent Artifacts in Single-Cell Genomics Studies. Genome Biol..

[64] Schaum N., Karkanias J., Neff N.F., May A.P., Quake S.R., Wyss-Coray T., Darmanis S., Batson J., Botvinnik O., Chen M.B. (2018). Single-Cell Transcriptomics of 20 Mouse Organs Creates a Tabula Muris. Nature.

[65] Muhl L., Genové G., Leptidis S., Liu J., He L., Mocci G., Sun Y., Gustafsson S., Buyandelger B., Chivukula I.V. (2020). Single-Cell Analysis Uncovers Fibroblast Heterogeneity and Criteria for Fibroblast and Mural Cell Identification and Discrimination. Nat. Commun..

[66] Walmsley G.G., Rinkevich Y., Hu M.S., Montoro D.T., Lo D.D., McArdle A., Maan Z.N., Morrison S.D., Duscher D., Whittam A.J. (2015). Live Fibroblast Harvest Reveals Surface Marker Shift In Vitro. Tissue Eng. Part C Methods.

[67] Philippeos C., Telerman S.B., Oulès B., Pisco A.O., Shaw T.J., Elgueta R., Lombardi G., Driskell R.R., Soldin M., Lynch M.D. (2018). Spatial and Single-Cell Transcriptional Profiling Identifies Functionally Distinct Human Dermal Fibroblast Subpopulations. J. Investig. Dermatol..

[68] Tabib T., Morse C., Wang T., Chen W., Lafyatis R. (2018). SFRP2/DPP4 and FMO1/LSP1 Define Major Fibroblast Populations in Human Skin. J. Investig. Dermatol..

[69] Guerrero-Juarez C.F., Dedhia P.H., Jin S., Ruiz-Vega R., Ma D., Liu Y., Yamaga K., Shestova O., Gay D.L., Yang Z. (2019). Single-Cell Analysis Reveals Fibroblast Heterogeneity and Myeloid-Derived Adipocyte Progenitors in Murine Skin Wounds. Nat. Commun..

[70] Buechler M.B., Pradhan R.N., Krishnamurty A.T., Cox C., Calviello A.K., Wang A.W., Yang Y.A., Tam L., Caothien R., Roose-Girma M. (2021). Cross-Tissue Organization of the Fibroblast Lineage. Nature.

[71] Rudnicka L., Varga J., Christiano A.M., Iozzo R.V., Jimenez S.A., Uitto J. (1994). Elevated Expression of Type VII Collagen in the Skin of Patients with Systemic Sclerosis. Regulation by Transforming Growth Factor-Beta. J. Clin. Investig..

[72] Tarhan L., Bistline J., Chang J., Galloway B., Hanna E., Weitz E. (2023). Single Cell Portal: An Interactive Home for Single-Cell Genomics Data. bioRxiv.

[73] Baysoy A., Bai Z., Satija R., Fan R. (2023). The Technological Landscape and Applications of Single-Cell Multi-Omics. Nat. Rev. Mol. Cell Biol..

[74] Foster D.S., Januszyk M., Yost K.E., Chinta M.S., Gulati G.S., Nguyen A.T., Burcham A.R., Salhotra A., Ransom R.C., Henn D. (2021). Integrated Spatial Multiomics Reveals Fibroblast Fate during Tissue Repair. Proc. Natl. Acad. Sci. USA.

[75] Hu J., Schroeder A., Coleman K., Chen C., Auerbach B.J., Li M. (2021). Statistical and Machine Learning Methods for Spatially Resolved Transcriptomics with Histology. Comput. Struct. Biotechnol. J..

[76] Vahid M.R., Brown E.L., Steen C.B., Zhang W., Jeon H.S., Kang M., Gentles A.J., Newman A.M. (2023). High-Resolution Alignment of Single-Cell and Spatial Transcriptomes with CytoSPACE. Nat. Biotechnol..

